# Regulatory Mechanisms of Autophagy-Targeted Antimicrobial Therapeutics Against Mycobacterial Infection

**DOI:** 10.3389/fcimb.2021.633360

**Published:** 2021-03-22

**Authors:** Prashanta Silwal, Seungwha Paik, Jin Kyung Kim, Tamotsu Yoshimori, Eun-Kyeong Jo

**Affiliations:** ^1^ Department of Microbiology, Chungnam National University School of Medicine, Daejeon, South Korea; ^2^ Infection Control Convergence Research Center, Chungnam National University School of Medicine, Daejeon, South Korea; ^3^ Department of Genetics, Graduate School of Medicine, Osaka University, Osaka, Japan; ^4^ Department of Intracellular Membrane Dynamics, Graduate School of Frontier Biosciences, Osaka University, Osaka, Japan

**Keywords:** autophagy, AMPK, mTOR, *Mycobacterium tuberculosis*, host-directed therapeutics

## Abstract

*Mycobacterium tuberculosis* (Mtb) is an intracellular pathogen causing human tuberculosis, an infectious disease that still remains as a global health problem. Autophagy, a lysosomal degradative process, has emerged as a critical pathway to restrict intracellular Mtb growth through enhancement of phagosomal maturation. Indeed, several autophagy-modulating agents show promise as host-directed therapeutics for Mtb infection. In this Review, we discuss recent progress in our understanding the molecular mechanisms underlying the action of autophagy-modulating agents to overcome the immune escape strategies mediated by Mtb. The factors and pathways that govern such mechanisms include adenosine 5′-monophosphate-activated protein kinase, Akt/mammalian TOR kinase, Wnt signaling, transcription factor EB, cathelicidins, inflammation, endoplasmic reticulum stress, and autophagy-related genes. A further understanding of these mechanisms will facilitate the development of host-directed therapies against tuberculosis as well as infections with other intracellular bacteria targeted by autophagic degradation.

## Introduction

Autophagy is an intracellular degradation process that maintains cell homeostasis during stress conditions (Ryter et al., 2013). Autophagy process is linked to various biological responses, including inflammation, metabolism, and innate effector pathways (Gutierrez et al., 2004; Deretic, 2008; Rabinowitz and White, 2010; Kimmey et al., 2015; Bergman et al., 2020; Chai et al., 2020; Mendes et al., 2020; Painter et al., 2020). *Mycobacterium tuberculosis* (Mtb) is the causal pathogen of human tuberculosis, a serious infectious disease with an increasing burden of drug resistance (Zumla et al., 2015; WHO, 2020). Autophagy functions as a cell-autonomous defensive pathway against intracellular Mtb (Gutierrez et al., 2004; Kimmey et al., 2015). After phagocytosis, a majority of Mtb resides in phagosome to escape the phagolysosomal acidification, but some of them access to cytosol and can be targeted by xenophagy (Watson et al., 2012; Manzanillo et al., 2013; Gomes and Dikic, 2014; Paik et al., 2019). Indeed, a variety of agents triggering autophagy/xenophagy promote phagosomal maturation through autophagic capture of either intraphagosomal Mtb or cytosolic pathogens (Gupta et al., 2016; Kim Y. S. et al., 2019).

In this Review, we outline the mechanisms underlying the effects of autophagy-based agents to enhance host defense against Mtb infection. In particular, we discuss the mechanisms and signaling pathways (adenosine 5′-monophosphate [AMP]-activated protein kinase [AMPK], mammalian target of rapamycin [mTOR] kinase, Wnt, transcription factor EB [TFEB], cathelicidins, inflammation, endoplasmic reticulum [ER] stress, and autophagy-related genes [ATGs]) that would make autophagy-activating agents a potential host-directed therapeutic (HDT) or alternative to current tuberculosis (TB) chemotherapeutics.

## Overview of Autophagy During Mycobacterial Infection

Autophagy is a catabolic process of damaged cellular components to ensure cell survival and homeostasis (Glick et al., 2010; Ryter et al., 2013). There are three canonical autophagy pathways—macroautophagy, microautophagy, and chaperone-mediated autophagy, which differ in how the cargo is targeted and delivered to lysosomes (Glick et al., 2010; Ryter et al., 2013). Macroautophagy (hereafter referred to as autophagy) is activated by stress signals including starvation, hypoxia, and infections, and is characterized by the formation of double-membraned autophagosomes, which fuse with a lysosome to form an autolysosome, the site of cargo degradation (Glick et al., 2010; Ryter et al., 2013).

Mtb has developed numerous strategies to avoid autophagic defense and manipulate host innate immunity (Jiao and Sun, 2019). For example, *via* the ESX-1 system, Mtb suppresses the late-stage autophagy in human dendritic cells to escape dendritic cell-mediated immunity (Romagnoli et al., 2012). The enhanced intracellular survival (Eis) gene of Mtb inhibits macrophage autophagy, at least partly mediated through suppression of c-Jun N-terminal kinase (JNK)-reactive oxygen species (ROS) signaling, in macrophages (Shin et al., 2010a). Also, Mtb lipoprotein LprE inhibits autophagy and cathelicidin expression to favor bacterial replication during infection (Padhi et al., 2019). In addition, virulent Mtb strains inhibit the recruitment of Rab7, the late endosomal/lysosomal protein, to the phagosomes, thereby escaping from phagosomal fusion with lysosomes (Chandra et al., 2015; Chandra and Kumar, 2016). However, ATGs, except ATG5, in myeloid cells do not appear to be essential in the activation of host defense *in vivo* (Kimmey et al., 2015). In addition, Mtb pathogens can epigenetically control host autophagy pathway through regulation of microRNAs (miRNAs) to favor mycobacterial replication in the host cells during infection (Batista et al., 2020; Ruiz-Tagle et al., 2020; Silwal et al., 2020). The miRNAs that are associated with pathogenesis of Mtb infection include miR-33/miR-33* (Ouimet et al., 2016), miR-889 (Chen et al., 2020), miR-18a (Yuan et al., 2020), and miR-125a (Kim et al., 2015), all of which are increased by Mtb infection; whereas others such as miR-26a (Sahu et al., 2017) and miR-17-5p (Kumar et al., 2016), both of which are decreased by Mtb infection. Numerous miRNAs that are involved in the regulation of autophagy in terms of host-pathogen interaction during Mtb infection have been extensively discussed elsewhere (Kim J. K. et al., 2017; Sabir et al., 2018; Yang and Ge, 2018; Silwal et al., 2020; Sinigaglia et al., 2020) and are not the focus of this Review. Thus, it remains to be fully characterized the exact mechanisms by which Mtb evade from host autophagic defense system, although several autophagy-activating drugs/agents are able to suppress Mtb growth *in vitro* and *in vivo* (Stanley et al., 2014; Gupta et al., 2016; Paik et al., 2019).

Noncanonical autophagy includes selective autophagy processes, such as xenophagy (intracellular bacteria) (Chai et al., 2019), mitophagy (damaged mitochondria) (Lazarou et al., 2015), lipophagy (lipid droplets) (Zhao et al., 2020), and etc. The ESX-1 system of Mtb is responsible for phagosomal damage and cytoplasmic release of bacteria and its ubiquitination, recruiting autophagic adaptors such as p62 and NDP52 to activate xenophagy (Manzanillo et al., 2013; Kim Y. S. et al., 2019). During xenophagy process, the ubiquitin ligases Parkin and Smurf1 are involved in the ubiquitination of cytosolic Mtb (Manzanillo et al., 2013; Franco et al., 2017). The DNA sensor cGAS triggers xenophagy through recognition of cytosolic Mtb DNA (Watson et al., 2015). Recent study showed that ubiquitin directly binds to the Mtb surface protein Rv1468c, triggering p62-mediated xenophagy (Chai et al., 2019). Advances in the identification on Mtb effector(s) to activate or inhibit xenophagy will elucidate the precise molecular mechanisms underlying Mtb manipulation of host autophagic process.

**Figure 1:**
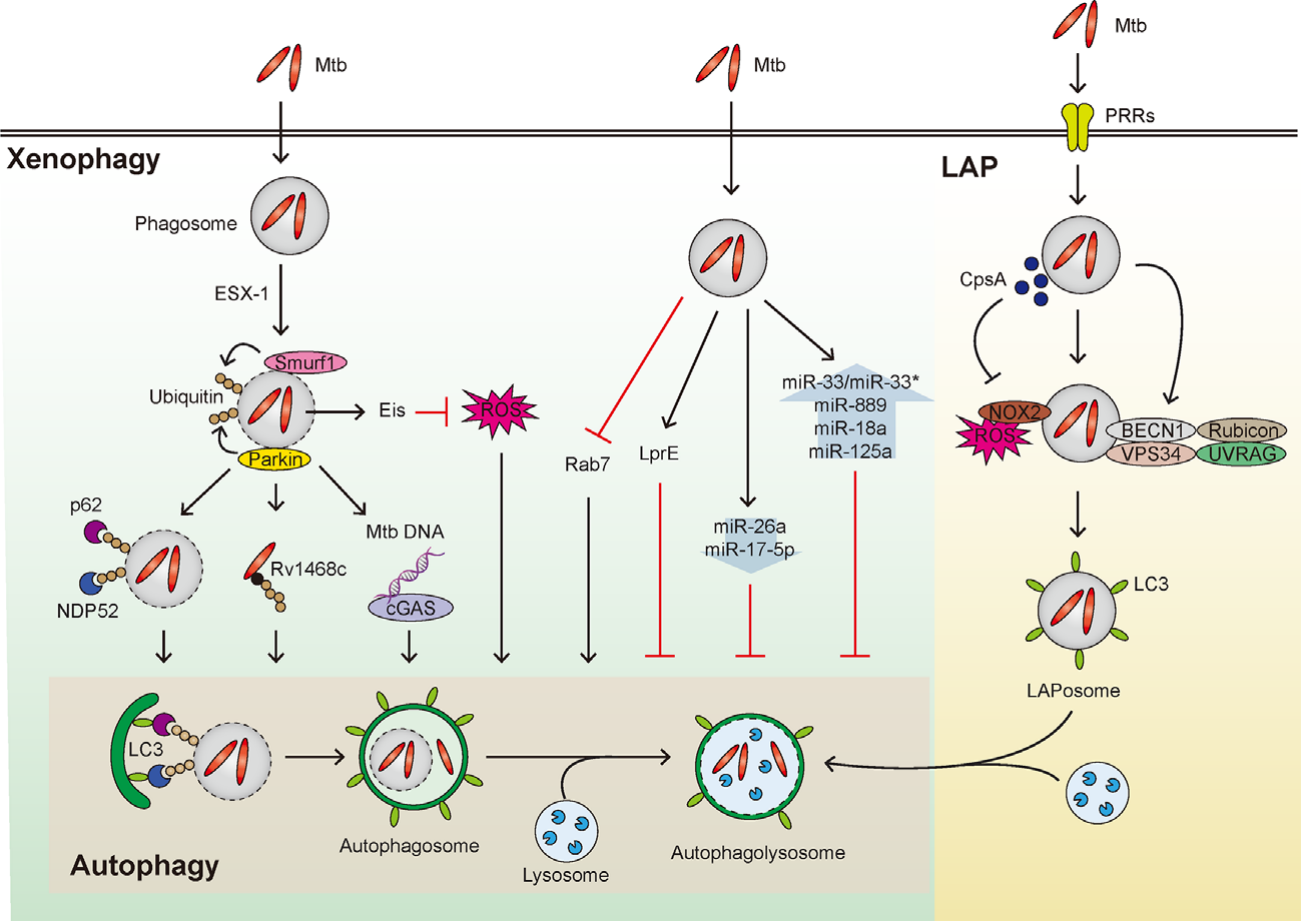
Overview of xenophagy and LAP during Mtb infection. Xenophagy and LAP are activated in host cells during Mtb infection. (Left) Mtb that enters the host cells by phagocytosis can reside within phagosomes and resist fusion with lysosomes. The phagosome is then damaged by the ESX-1 system to induce Smurf1- and Parkin-mediated ubiquitination, thereby adaptor proteins such as p62 and NDP52 are recruited leading to formation of autophagosome. Also, Rv1468c, a surface protein of Mtb that escapes from the phagosome, induces xenophagy by binding with ubiquitin. In addition, the Mtb DNA exposed to the cytosol from the damaged phagosome is recognized by cGAS to induce xenophagy. Both the enhanced intracellular survival (Eis) gene of Mtb and putative Mtb lipoprotein LprE inhibit autophagy. Additionally, several miRNAs that inhibit xenophagy are shown in the context of mycobacterial infection. In addition, Mtb inhibits the recruitment of Rab7, thus disturbing the fusion of autophagosome and lysosome. (Right) Various PRRs recognize Mtb and invade the host cell. Rubicon complex (Rubicon-BECN1-VPS34-UVRAG) and NOX2-dependent ROS are important in the activation of LAP. In this process, LAPosome, a single membrane surrounded by LC3 is formed, which fuses with lysosome to eliminate Mtb. At this time, it is known that CpsA, a protein of Mtb, interferes with the LAP mechanism.

In addition, LC3-associated phagocytosis (LAP), another form of noncanonical autophagy (Sanjuan et al., 2009), appears to be essential for host defense against mycobacterial infection (Koster et al., 2017). LAP links between pattern recognition receptor (PRRs) signaling and phagosomal maturation, and is characterized by a single-membrane phagosomal structure (Sanjuan et al., 2009; Sprenkeler et al., 2016). Unlike canonical autophagy, LAP does not require pre-initiation of the autophagy machinery complex (Sanjuan et al., 2009; Sprenkeler et al., 2016), but makes use of Rubicon and NADPH oxidase 2 (NOX2) (Martinez et al., 2015). The mycobacterial protein CpsA protects Mtb from NOX2-dependent ROS and subsequent LAP-mediated killing (Koster et al., 2017). It is warranted to clarify how Mtb and its products orchestrate LAP function to escape from host anti-mycobacterial responses. Mtb-induced regulation of xenophagy and LAP is summarized in **Figure 1**.

So far, numerous agents/small molecules to promote host anti-mycobacterial defense through targeting autophagy have been discussed (Palucci and Delogu, 2018; Singh and Subbian, 2018; Kim Y. S. et al., 2019; Paik et al., 2019). In this Review, we focus on recent advances of several mechanisms by which autophagy-targeting startegies enhance host defense against mycobacterial infections.

## AMPK and Innate Host Defense Against Mtb

AMPK, a key metabolic regulator, plays a role in the regulation of autophagy during a variety of stress conditions. Pharmacological activation of AMPK has therapeutic potential for a variety of human diseases, including ischemia-reperfusion (I/R) injuries, cardiovascular diseases, and bone-related diseases (Ding et al., 2020; Li et al., 2020; Tong et al., 2020). In addition, the AMPK pathway is involved in the antimicrobial response to Mtb infection (Silwal et al., 2018). The AMPK activator AICAR and the mycobacterial lipoprotein LpqH are able to activate autophagy in macrophages *via* AMPK signaling pathway (Shin et al., 2010b; Yang et al., 2014). A recent study suggest a potential role for TNF-like weak inducer of apoptosis (TWEAK) in AMPK-mediated autophagy activation during mycobacterial infection. TWEAK levels are upregulated in human peripheral blood mononuclear cells by stimulation with heat-killed Mtb or secreted proteins of Mtb, and in sera from TB patients at the early phase, but declined in patients with latent tuberculosis infection (LTBI) (Chen et al., 2020). TWEAK activates autophagy and phagosomal maturation in macrophages through activation of AMPK (Chen et al., 2020). Interestingly, the increased miR-889 level in LTBI suppresses autophagy through targeting TWEAK; and after TNF-α inhibitor therapy, TWEAK level decreases, thereby contributing to LTBI reactivation in rheumatoid arthritis patients with LTBI (Chen et al., 2020).

Mtb infection increases miR-18a expression to suppress the autophagy; and miR-18a inhibition increases AMPK phosphorylation and promotes autophagy, resulting in suppression of intracellular Mtb survival (Yuan et al., 2020). Mtb induction of miR33/33* supports intracellular bacterial replication by inhibiting the autophagic flux *via* repression of AMPK-dependent activation of forkhead box O (FOXO) 3 and TFEB (Ouimet et al., 2016). In addition, AMPK activation by AICAR or resveratrol induces transcriptional and post-translational activation of autophagy genes *via* orphan nuclear receptor estrogen-related receptor (ERR)-α (Kim S. Y. et al., 2018). These data suggest that AMPK activation can be regulated transcriptionally or epigenetically in the context of the autophagy activation. Further studies are needed to elucidate the exact molecular pathways through which AMPK-induced autophagy activation contributes to host defense against Mtb infection.

Recent studies have highlighted the roles of metabolites in the activation of autophagy and host defense through AMPK signaling (Kim J. K. et al., 2018; Sivangala Thandi et al., 2020). A metabolite ornithine is implicated in Kupffer cell-specific host defense against Mtb infection. Ornithine, a product of Mtb-infected Kupffer cells, induces autophagy through AMPK, thereby restricting Mtb growth (Sivangala Thandi et al., 2020). The neurotransmitter and metabolite gamma-aminobutyric acid (GABA) triggers autophagy by activating AMPK through calcium flux, and leads to enhanced innate host defense against Mtb infection (Kim J. K. et al., 2018). Although not confirmed in human trials, these preclinical observations suggest the utility of autophagy-targeting metabolites for HDTs against TB. Autophagy-based antimicrobial responses acting through AMPK are summarized in **Table 1**.

**Table 1:** Autophagy-based antimicrobial responses acting through AMPK and Akt/mTOR pathways.

Activator	Study model	Pathogen	Mechanism	Ref.
**AMPK**				
AICAR	BMDMs, RAW264.7 & THP-1 cells	Mtb H37Rv	Upregulation of autophagy-related genes expression through AMPK-PPARGC1A pathway	(Yang et al., 2014)
AICAR	BMDMs	Mtb H37Rv	Induction of AMPK/SIRT1-mediated ESSRA to enhance the transcriptional and post-transcriptional activation of autophagy genes	(Kim S. Y. et al., 2018)
LpqH	Human primary monocytes	Mtb H37Rv	Induction of TLR2/1/CD14-mediated C/EBP-β-dependent CYP27B1 hydrolase and cathelicidin expression, Activation of Ca^2+^/AMPK/p38 MAPK signaling pathway	(Shin et al., 2010b)
TWEAK	THP-1 cells	*M. bovis* BCG	Promotion of mycobacterial phagosomal maturation through AMPK activation	(Chen et al., 2020)
miR-18a inhibitor	RAW264.7 cells	Mtb H37Rv	Regulation of ATM-AMPK pathway	(Yuan et al., 2020)
miR-33/miR-33* inhibitor	PMs	Mtb H37Rv	Activation of AMPK-dependent FOXO3 and TFEB	(Ouimet et al., 2016)
Ornithine	Mouse alveolar macrophages, Kupffer cells	Mtb H37Rv	Reduction of ammonia and induction of AMPK	(Sivangala Thandi et al., 2020)
GABA	BMDMs	Mtb H37Rv	Activation of GABA_A_R-Ca^2+^-AMPK signaling, GABARAPL1-mediated phagosomal maturation	(Kim J. K. et al., 2018)
**Akt/mTOR**				
Bazedoxifene	THP-1 cells	Mtb H37Ra	Induction of ROS production and inhibition of Akt/mTOR signaling	(Ouyang et al., 2020)
Nilotinib	BMDMs, RAW264.7 cells	*M. bovis*	Inhibition of cABL to induce Akt/mTOR mediated autophagy and parkin mediated xenophagy	(Hussain et al., 2019)
Baicalin	RAW264.7 cells, PMs	Mtb H37Ra	Inhibition of Akt/mTOR as well as Akt/NF-κB pathway, Inhibition of NLRP3 inflammasome and IL-1β	(Zhang et al., 2017)
Isoniazid-incorporated Man-Se NPs	THP-1 cells	Mtb H37Rv, *M. bovis* BCG	Inhibition of PI3K/Akt/mTOR signaling, Upregulation of ROS production and inhibition of mitochondrial function	(Pi et al., 2020)
Rapamycin	MSCs, THP-1 cells	Mtb H37Rv	Elimination of actively replicating and latent bacteria with the combination of antibiotics and rapamycin	(Fatima et al., 2020)
Rapamycin microparticles	THP-1 cells	Mtb H37Rv	Increased uptake of rapamycin loaded PLGA particles targeting macrophages	(Gupta et al., 2014)

BMDMs, Bone marrow derived macrophages; Mtb, Mycobacterium tuberculosis; M. bovis BCG, Mycobacterium bovis bacillus Calmette-Guerin (BCG); MDMs, Monocyte derived macrophages; AMPK, AMP-activated protein kinase; PPARGC1A, Peroxisome proliferator-activated receptor-gamma, coactivator 1α; SIRT1, Sirtuin 1; ESSRA, Estrogen related receptor alpha; TLR, Toll-like receptor; C/EBP-β, CCAAT/enhancer-binding protein beta; CYP27B1, Cytochrome p450 27B1; MAPK, Mitogen-activated protein kinase; ATM, Ataxia telangiectasia mutated; FOXO3, Forkhead box O3; PMs, Peritoneal macrophages; GABA, Gamma-aminobutyric acid; GABA_A_R, GABA_A_ Receptor; ROS, Reactive oxygen species; mTOR, Mammalian target of rapamycin; cABL, Ableson tyrosine kinase ABL; NLRP3, NLR family pyrin domain containing 3; NPs, Nanoparticles; MSCs, Human mesenchymal stem cells; PLGA, Poly(lactide-co-glycolide).

## The mTOR/Akt Pathway and Host Defense Against Mtb

mTOR is a serine/threonine protein kinase consisting of mTORC1 and mTORC2 (Jhanwar-Uniyal et al., 2019), and regulates a variety of biological processes, including protein synthesis, cell proliferation, growth, autophagy, and metabolism (Gibbons et al., 2009; Kaur and Sharma, 2017; Qian et al., 2020). mTORC1 is regulated by the phosphatidylinositol 3-kinase (PI3K)/Akt pathway, constitutive activation of which suppresses autophagy process (Kaleagasioglu et al., 2020). Earlier studies showed that Mtb and its components can trigger the signaling pathways of PI3K/Akt and mTOR/S6K1 in macrophages (Maiti et al., 2001; Yang et al., 2006; Yang et al., 2014). Thus, it is possible that Mtb-mediated mTOR/Akt signaling may play a major role for suppression of macroautophagy during infection (Yang et al., 2006; Yang et al., 2014). Rapamycin, a suppressor of mTORC1, is a classical activator of autophagy (Li et al., 2014), shows an antimicrobial effect upon intracellular Mtb replication, as reported in earlier study (Gutierrez et al., 2004). Because novel analogs of rapamycin (temsirolimus, everolimus, and deforolimus) have therapeutic potential through activation of autophagy in the context of various diseases including cancers (Yazbeck et al., 2008; Gibbons et al., 2009; Cerni et al., 2019; Zou et al., 2020), it is likely that these mTOR inhibitors have beneficial effects for antibacterial autophagy and host defense against Mtb infection. Future preclinical and clinical studies will clarify the precise role for several candidates of rapamycin analogs as a promising target for autophagy-based therapeutics against Mtb infection as well as other infectious diseases.

Indeed, pharmacological inhibitors of Akt/mTOR signaling, through regulation of autophagy, have emerged as promising approaches for therapeutics against cancers or liver diseases (Wang et al., 2019; Kaleagasioglu et al., 2020). Recent studies by using several drugs/agents that inhibit mTOR/Akt pathway have shed a light on autophagy-based antimicrobial therapy against Mtb or nontuberculous mycobacteria (NTM) infection. Bazedoxifene, a selective estrogen receptor modulator, promotes autophagy to suppress intracellular growth of Mtb through upregulation of ROS production and inhibition of Akt/mTOR signaling (Ouyang et al., 2020). Nilotinib, a tyrosine kinase inhibitor, induces xenophagy against *M*. *bovis* infection by inhibiting the PI3K/Akt/mTOR axis (Hussain et al., 2019). In addition, the herbal agent, baicalin, suppresses intracellular Mtb by inducing autophagy in murine macrophages *via* inhibition of Akt and mTOR phosphorylation (Zhang et al., 2017). However, targeting the mTORC1 pathway in patients co-infected with Mtb and human immunodeficiency virus (HIV) requires caution, because mTOR inhibition reduced phagosomal acidification and led to increased Mtb replication in co-infected conditions (Andersson et al., 2016). So far, most attempts by using mTOR/Akt inhibitors focus on cancers (Kaleagasioglu et al., 2020). Future studies are needed to explore the potential or limitation of mTOR/Akt-targeted therapeutics against infections with antibiotic resistant Mtb or NTM. As cancers and Mtb infection share similar metabolic profiles (Shi et al., 2015; Gleeson et al., 2016), embracing an approach based on immunometabolic assessment will be crucial to develop future therapeutics which incorporate the inhibitors of Akt/mTOR signaling pathway, a major metabolic regulator (Covarrubias et al., 2015), into anti-mycobacterial activity evaluation.

Isoniazid-conjugated selenium (Se) nanoparticles (NPs) promote the fusion of Mtb to lysosomes and exert a synergistic effect on lysosomal and isoniazid-induced destruction of Mtb in macrophages (Pi et al., 2020). Mechanistically, isoniazid-conjugated Man-Se NPs promote autophagic degradation of Mtb through generation of intracellular ROS and inhibition of PI3K/Akt/mTOR pathway (Pi et al., 2020). Additionally, autophagy activation by rapamycin contribute to the elimination of Mtb in mesenchymal stem cells, which are important for dormancy (Fatima et al., 2020). Moreover, treatment of rapamycin, combined with isoniazid, promote sterile clearance and prevention of TB reactivation *in vivo* (Fatima et al., 2020). Together with the efficacy of inhalable rapamycin in clearing Mtb (Gupta et al., 2014), these findings suggest mTOR signaling pathway could be novel targets for adjunctive HDT against TB latency and reactivation. Autophagy-based antimicrobial responses acting through mTOR/Akt are summarized in **Table 1**.

## Wnt Signaling Pathway

The Wnt/β-catenin pathway, which is important for cell proliferation and polarity, is a target of bacterial virulence factors of invading intracellular pathogens, including Mtb (Villasenor et al., 2017; Ljungberg et al., 2019; Silva-Garcia et al., 2019). Meanwhile, the inhibitors of the Wnt/β-catenin pathway used to treat cancers (Yan et al., 2017; Liu et al., 2019; Silva-Garcia et al., 2019) may have potential to control the pathological responses during chronic Mtb infection (Brandenburg and Reiling, 2016). However, it is just in its infancy to understand the precise roles of Wnt signaling in the context of autophagy pathway during mycobacterial infection.

Several studies reported that the Wnt signaling is linked to immune responses and autophagy pathways in terms of intracellular mycobacterial infection. The IL-1 family cytokine IL-36γ suppresses intracellular survival of Mtb by WNT5A-dependent activation of autophagy in human monocyte-derived macrophages (MDMs) (Gao et al., 2019). IL-36γ-mediated autophagy activation is dependent on WNT5A expression in noncanonical Wnt pathway by regulating COX-2/Akt/mTOR axis (Gao et al., 2019). In contrast, LKB1 inhibits intracellular Mtb by downregulating WNT5A and upregulating FOXO1 expression in human macrophages (Cui et al., 2019). Another study shows that Wnt3a, a ligand of Wnt signaling, suppresses BCG-induced autophagy through mTOR-dependent pathway and the expression of autophagy proteins such as LC3 in macrophages (Wu et al., 2019). Together, it has not been fully understood the clear role and mechanisms underlying Wnt signaling-mediated autophagy in regulating antibacterial host defense against Mtb infection. Given the multiple Wnt signaling molecules may play various roles in the different context of infection (Ljungberg et al., 2019; Rogan et al., 2019), future studies are warranted to understand the exact molecular mechanisms by which specific Wnt ligands and modulators regulate autophagy-mediated anti-mycobacterial activities *in vivo* as well as *in vitro*.

## TFEB

Several transcription factors—such as TFEB, FOXO, and nuclear factor erythroid 2-related factor (NRF)—are downstream molecules of mTOR signaling (Schmeisser and Parker, 2019). Among them, TFEB, a member of the microphthalmia/transcription factor E (MiT-TFE) family of basic helix-loop-helix leucine-zipper transcription factors, is a key factor for lysosome biogenesis, energy homeostasis, and autophagy (Napolitano and Ballabio, 2016; Puertollano et al., 2018). The mTOR/Akt pathway regulates subcellular localization of TFEB by phosphorylating TFEB and binding to 14-3-3 protein, thereby inhibiting its nuclear translocation and activation (Roczniak-Ferguson et al., 2012; Napolitano and Ballabio, 2016; Puertollano et al., 2018). Interferon (IFN)-γ-induced autophagy is mediated through intracellular calcium-triggered activation of the phosphatase calcineurin (PPP3)-TFEB pathway, thus inhibiting mycobacterial survival in macrophages (Singh et al., 2018). PPARα agonists promote autophagy and lysosomal gene activation (including TFEB) to enhance anti-mycobacterial defense (Kim Y. S. et al., 2017). In addition, TFEB controls excessive inflammatory responses during Mtb infection, because silencing of TFEB promotes proinflammatory cytokine synthesis in macrophages (Kim Y. S. et al., 2017). Furthermore, sirtuin 3 enhances antibacterial autophagy through TFEB expression during mycobacterial infection (Kim et al., 2019). The sirtuin 3 deficiency exaggerates immune pathology and *in vivo* bacterial burden during Mtb infection (Kim T. S. et al., 2019; Smulan et al., 2021). Sirtuin 3 activation by honokiol enhances antimicrobial responses through activation of antibacterial autophagy and amelioration of mitochondrial oxidative stress during Mtb infection (Kim T. S. et al., 2019). However, it is unclear whether sirutin 3-TFEB-mediated anti-inflammatory responses are directly associated with the host defense against Mtb.

Some antibiotics including linezolid and bedaquiline (BDQ) activate autophagy to promote clearance of Mtb (Genestet et al., 2018; Giraud-Gatineau et al., 2020). BDQ induces autophagy by upregulating lysosomal activation *via* TFEB and calcium signaling and potentiates the activity of other anti-TB drugs (Giraud-Gatineau et al., 2020). Ambroxol, a mucolytic agent identified by screening of autophagy-inducing drugs, promotes antimicrobial responses by inducing autophagy *via* nuclear translocation of TFEB (Choi S. W. et al., 2018). Together, these data suggest that TFEB plays a critical function in the activation of anti-mycobacterial responses to a variety of autophagy-activating agents and could be a principal target for developing autophagy-based therapeutics against TB.

## Antimicrobial Proteins: Cathelicidins

Cathelicidin functions as an antimicrobial peptide and a second messenger in vitamin D-mediated antimicrobial responses, inflammation, and autophagy during Mtb infection (Jo, 2010; Chung et al., 2020). The expression of cathelicidin antimicrobial peptide (CAMP)/LL-37 and ATG expression in human macrophages is inhibited by Mtb (Rekha et al., 2015). A recent study shows that Mtb LprE suppresses cathelicidin expression *via* p38 MAPK pathway and inhibit autophagy in macrophages (Padhi et al., 2019). Indeed, the CAMP-mediated autophagy activation is importantly implicated in vitamin D-mediated host defense during Mtb infection (Liu et al., 2007; Yuk et al., 2009; Fabri et al., 2011; Rekha et al., 2015; Chung et al., 2020). Several innate immune signals including TLR2/1, TLR8, and cytokines are linked to regulation of cathelicidin/autophagy pathway to promote antimicrobial function (Shin et al., 2010b; Campbell and Spector, 2012; Yang et al., 2018; Padhi et al., 2019). Although these studies strongly suggest that cathelicidins play a critical role in autophagy as a second messenger, the exact mechanisms by which cathelicidins modulate the activation of autophagy are not clear.

Co-treatment of IL-12/IL-18 enhances anti-mycobacterial responses in human macrophages and lung epithelial cells in the context of IFN-γ-mediated immunity and the CAMP/autophagy pathway (Yang et al., 2018). Thus, cathelicidins may function as critical mediators to linking innate and adaptive immune responses during Mtb infection (Chung et al., 2020). In addition, several factors should be considered to elucidate the host defensive function of cathelicidins during Mtb infection. For example, prostaglandin E2 (PGE2), a lipid mediator, impairs vitamin D3-induced cathelicidin expression and autophagy, thus promoting growth of Mtb in macrophages (Wan et al., 2018). Because vitamin D insufficiency is associated with susceptibility to TB (Talat et al., 2010; Pilarski et al., 2016; Nouri-Vaskeh et al., 2019), further knowledge on clinical application should be accumulated to clarify the efficacy of cathelicidins as adjuvants in HDTs against TB.

## Inflammation

Suppression of inflammation and tissue damage by modulating the host immune response are achieved through several modalities of HDTs against TB (Ahmed et al., 2020). During Mtb infection, inflammatory pathway is linked to autophagic defense in various ways. The combination of vitamin D3 and phenylbutyrate significantly decreases the levels of proinflammatory cytokines/chemokines in peripheral blood mononuclear cells (PBMCs) and increases the frequency of autophagy thereby exerting a favorable immunomodulatory effect (Rekha et al., 2018). Also, the herbal agent baicalin, an inducer of autophagy, inhibits NF-κB signaling pathway and NLRP3 inflammasome activation, exerting an anti-inflammatory and antimicrobial effect (Zhang et al., 2017).

Although autophagy counteracts excessive inflammatory responses (Qian et al., 2017), its activation by lipopolysaccharide (LPS) restores Mtb-inhibited IL-12 production, promoting protective immunity (Fang et al., 2020). In addition, the C-type lectin receptor CLEC4E in association with TLR4 agonists (C4.T4) significantly improves antimicrobial host defense by activating MyD88-dependent autophagy and lysosomal biogenesis (Pahari et al., 2020). Importantly, C4.T4 agonists in combination with isoniazid or rifampicin kill intracellular Mtb to improve the efficacy of anti-TB drugs at lower dose than that of currently in use (Pahari et al., 2020). These studies suggest that, either by controlling excessive inflammation or inducing innate immune responses, activation of host-cell autophagy may contribute to the efficacy of anti-TB drugs during TB treatment. Future studies are needed to understand the exact mechanisms by which autophagy-targeting drugs/agents shape the profile of inflammatory responses during Mtb infection to impact host defensive function.

## Endoplasmic Reticulum Stress and Mycobacterial Infection

ER homeostasis is mediated by the unfolded protein response (UPR), which involves IRE1/sXBP1, PERK/EIf2, and ATF6 (Tameire et al., 2015). Dysregulation of ER homeostasis is associated with ER stress and leads to various diseases including autoimmune diseases and skin related pathologies (Junjappa et al., 2018; Park et al., 2019). The ER stress response is linked to innate immune signaling, lipid accumulation, and proinflammatory responses in myeloid-lineage cells during infection and inflammation (Martinon and Glimcher, 2011; Yao et al., 2014; Di Conza and Ho, 2020; Sukhorukov et al., 2020). Mtb and its components trigger ER stress to modulate host cell death, intracellular survival, and inflammatory responses (Lim et al., 2016; Jo et al., 2017; Lee et al., 2019). Indeed, activation of autophagy by the ER stress response is related to cytoprotection, particularly in tumor cells (Rouschop et al., 2010; Hart et al., 2012; Bhardwaj et al., 2019). It is warranted to study whether regulation of autophagy in the connection with UPR and/or ER stress may also exert a beneficial effect in modulating the pathological responses induced by Mtb infection.

ER stress and autophagy are linked together during Mtb infection, although the precise molecular mechanisms are largely unknown. BAG2 (BCL2-associated athanogene 2), a protein associated with cell fate determination, promotes autophagic flux and reticulophagy by enhancing SQSTM1/p62 localization to the ER and contributes to host cell resistance to Mtb-mediated damage (Liang et al., 2020). Mechanistically, BAG2-induced autophagy activation involves ERK-mediated dissociation of Beclin-1 (BECN1)-BCL2 complex, and XBP1-induced transcriptional activation (Liang et al., 2020). These data suggest that BAG2 promotes antimicrobial defense against Mtb infection by linking ER stress and selective autophagy (Liang et al., 2020). In addition, ajoene, a garlic-derived sulfur-containing compound, exerts an anti-mycobacterial effect by activating autophagy and ER stress (Choi J. A. et al., 2018). Ajoene-mediated JNK activation and ROS production contributes to the activation of ER stress, autophagy, and anti-mycobacterial effects (Choi J. A. et al., 2018). These studies suggest that ER stress and autophagy pathways are interconnected for anti-mycobacterial host defense, yet many challenging questions remain about the cross talks between ER stress and autophagy, both are crucial in the maintenance of intracellular protein homeostasis (Rashid et al., 2015). Additional functional analyses are needed to elucidate the roles and molecular mechanisms underlying that ER stress interacts with autophagy pathway during mycobacterial infection.

## Miscellaneous and Unknown Mechanisms

Substantial evidences exist that several autophagy-activating agents effectively increase transcriptional activation of ATGs and nonspecific enhancement of autophagy. Phenotypic whole-cell screening using *M. bovis* BCG showed that 5-nitro-1,10-phenanthroline (5NP) inhibits intracellular Mtb, at least in part by activating autophagy and increasing BECN1 and ATG3 expression (Kidwai et al., 2017). In addition, the bovine antimicrobial peptide lactoferrin killed *M. avium* and enhanced the anti-mycobacterial effect of ethambutol in host cells by promoting formation of autophagosomes and lysosomes (Silva et al., 2017). Everolimus, a derivative of rapamycin, not only inhibits mTORC1 and promotes autophagy, but enhances immunity in elderly at low doses (Mannick et al., 2014), suggesting therapeutic potential for TB (Cerni et al., 2019). Future studies are needed to clarify whether everolimus at appropriate doses has a protective effect in host defense against Mtb infection, particularly in elderly, through activation of autophagy.

There is considerable interest in the development of combination treatment strategies based on autophagy-activating molecules/agents incorporating the currently used anti-TB drugs. Recent study showed that the pyrazole derivative small molecule, NSC 18725, inhibits growth of intracellular Mtb and exerts a synergistic effect with isoniazid (Arora et al., 2019). Also, NSC 18725-mediated activation of autophagy induces an antimicrobial response to intracellular Mtb and other fast-growing mycobacteria (Arora et al., 2019). However, the exact mechanism by which NSC 18725 induces autophagy and anti-mycobacterial effect is yet to be elucidated.

A recent study demonstrated that autophagy-activating agents have the adjuvant effect of vaccines. Curcumin NPs are promising adjuvants for TB vaccines, because curcumin NP-mediated antigen-presenting cell functions, including autophagy activation, contribute to induction of long-lasting central memory T cells by the BCG vaccine (Ahmad et al., 2019). Although the impact of autophagy in the development of adjuvants and vaccines is largely unknown, it is an exciting area of potentially high clinical relevance for prevention of TB. Autophagy-based antimicrobial responses targeting several mechanisms other than AMPK and mTOR pathways are summarized in **Table 2**.

**Table 2:** Autophagy-based antimicrobial responses against mycobacterial infections.

Target	Activator	Study model	Pathogen	Mechanism	Ref.
**WNT5A**	IL-36γ	Human MDMs	Mtb H37Rv, *M. bovis* BCG	Autophagy activation *via* COX-2/AKT/mTOR axis	(Gao et al., 2019)
	LKB1	THP-1 & U937 cells	Mtb H37Rv	Upregulation of FOXO1 expression	(Cui et al., 2019)
**Wnt3a**	–	RAW264.7 cells	*M. bovis* BCG	Autophagy inhibition by activating mTOR-dependent pathways	(Wu et al., 2019)
**TFEB**	IFN-γ	RAW264.7 cells, C57BL/6 mice	Mtb H37Rv	Nuclear translocation of TFEB through PPP3 in an HMOX1-dependent manner	(Singh et al., 2018)
	PPAR-α activation	BMDMs, C57BL/6 mice	Mtb H37Rv, *M. bovis* BCG	Promotion of TFEB-mediated autophagy	(Kim Y. S. et al., 2017)
	BDQ	Human MDMs	Mtb H37Rv	Upregulation of lysosmal activation *via* TFEB and calcium signaling	(Giraud-Gatineau et al., 2020)
	Ambroxol	BMDMs	Mtb Erdman	Induction of LC3B puncta and TFEB nuclear translocation	(Choi S. W. et al., 2018)
**Cathelicidins**	IL-12/IL-18	THP-1 & A549 cells, human MDMs	*M. bovis* BCG	Induction of VDR-derived cathelicidin and autophagy	(Yang et al., 2018)
	PGE2	MM6 cells, human MDMs	Mtb H37Rv	Inhibition of vitamin D3-induced cathelicidin and autophagy *via* EP2/EP4	(Wan et al., 2018)
**Inflammation**	Vitamin D+Phenylbutyrate	Human PBMCs	Mtb H37Rv	Reduction of proinflammatory cytokines/chemokines	(Rekha et al., 2018)
	LPS	THP-1 cells	Mtb H37Ra	Restoration of Mtb-inhibited IL-12 synthesis and secretion *via* autophagy activation	(Fang et al., 2020)
	C4.T4	BMDMs, C57BL/6 mice	Mtb H37Rv, H37Ra	Induction of autophagy *via* Myd88-dependent/mTOR-independent PI3K activation	(Pahari et al., 2020)
**ER Stress**	BAG2	BMDMs, RAW264.7 cells	Mtb H37Ra	Reduction of ER-stress-induced apoptosis through selective autophagy	(Liang et al., 2020)
	Ajoene	RAW264.7 cells	Mtb H37Rv	ER stress mediated ROS production and JNK activation	(Choi J. A. et al., 2018)
**Miscellaneous**	5NP	THP-1 cells	*M. bovis* BCG, *M. smegmatis*	Activation of autophagy with increased expression of BECN1 and ATG3	(Kidwai et al., 2017)
	Lactoferrin	BMDMs	*M. avium*	Increased formation of lysosomes and autophagosome-like vesicle	(Silva et al., 2017)
	NSC 18725	THP-1 cells	*M. bovis* BCG, *M. smegmatis*	Induction of autophagy with upregulation of BECN1 and ATG3	(Arora et al., 2019)
	Curcumin NPs	PMs, C57BL/6 mice	Mtb H37Rv, *M. bovis* BCG	Enhancement of autophagy and antigen presenting capacity of APCs	(Ahmad et al., 2019)

IL-36, Interleukin-36; MDMs, Monocyte derived macrophages; Mtb, Mycobacterium tuberculosis; M. bovis BCG, Mycobacterium bovis bacillus Calmette-Guerin (BCG); COX-2, Cyclooxygenase-2; LKB1, Liver kinase B1; FOXO1, Forkhead box protein O1; IFN, Interferon; TFEB, Transcription factor EB; PPP3, Protein phosphatase 3; HMOX1, Heme oxygenase 1; PPAR, Peroxisome proliferator-activated receptor; BMDMs, Bone marrow derived macrophages; BDQ, Bedaquiline; VDR, Vitamin D receptor; PGE2, Prostaglandin E2; EP, E prostanoid; PBMC, Peripheral blood mononuclear cells; C4.T4, Agonists of CLEC4E (C4/TDB) and TLR4 (T4/ultra-pure-LPS); Myd88, Myeloid differentiation primary response 88; PI3K, Phosphoinositide 3-kinases; BAG2, BCL2-associated athanogene 2; ER, Endoplasmic reticulum; JNK, c-Jun N-terminal kinases; 5NP, 5-Nitro-1,10-phenanthroline; NPs, Nanoparticles; PMs, Peritoneal macrophages; APC, Antigen presenting cells.

## Conclusion

The therapeutic and preventative efficacy of autophagy-activating agents for Mtb infection have been investigated extensively. As more agents/molecules activating the autophagy are identified, we face the challenge of understanding the underlying molecular mechanisms. These agents/molecules act through several mechanisms such as AMPK and mTOR pathways, TFEB, cathelicidins, inflammation, ER stress, and ATGs to ultimately modulate the autophagy processes and the anti-mycobacterial responses (**Tables 1** and **2**). Future studies will clarify whether many of those agents have the effects to increase the efficacy of anti-TB drugs in pre-clinical and clinical models. It is possible that several of these pathways function together and/or that key regulators of multiple signaling pathways cooperate in the activation of autophagy-targeted antimicrobial responses to Mtb infection. Such knowledge will facilitate development of therapeutics and vaccines for Mtb infection. In addition, the clinical relevance of *in vitro* findings on autophagy-based antimicrobial strategies against Mtb infection needs to be established.

## Author Contributions

E-KJ designed the study. E-KJ, TY, SP, JKK, and PS wrote and reviewed the manuscript. JKK prepared the figure; SP and PS summarized the tables. All authors contributed to the article and approved the submitted version.

## Funding

This work was supported by the National Research Foundation of Korea (NRF) grant funded by the Korea government (MSIT) (No.2019R1A2C1087686) and by the framework of international cooperation program managed by National Research Foundation of Korea (Grant Number:2015K2A2A6002008).

## Conflict of Interest

The authors declare that the research was conducted in the absence of any commercial or financial relationships that could be construed as a potential conflict of interest.
